# Postage Stamp Precision Technique of Dorsal Hump Reduction

**DOI:** 10.1055/s-0045-1802642

**Published:** 2025-03-24

**Authors:** Uday Bhat, Amit Peswani, Ishita Katyal, Anudeep T.C, Mangesh Pawar, Suparna P.N, Richa Goel

**Affiliations:** 1 Department of Plastic Surgery, Topiwala National Medical College and BYL Nair Charitable Hospital, Mumbai, Maharashtra, India; 2 Department of Plastic and Reconstructive Surgery, A La Mode Esthetique Studio, Mysore, Karnataka, India

**Keywords:** rhinoplasty, hump reduction, dorsal Hump

## Abstract

**Background** The conventional methods of dorsal hump reduction planning and execution are prone to errors. The amount to be lowered is usually subject to surgeon's judgment and errors are possible due to differential skin thickness of envelope.

**Objectives** We propose a hump reduction technique that includes a method to precisely plan the amount of excision based on real surface measurements and our postage stamp technique of hump reduction.

**Materials and Methods** A prospective study was done in 25 patients requiring dorsal hump reduction. The planning of hump reduction includes precisely plotting the extent of the hump from fixed landmarks on the skin and recreating these distances on the framework after raising the envelope. In contrast to the traditional continuous bony cut in caudocranial direction, we used serial perforations along the proposed profile line. These postage stamp cuts are made using 2 mm osteotome and are converted into a continuous cut using the double-guarded osteotome.

**Results** All 20 patients were followed till 1 year. There was no major complication and all patients were satisfied with the appearance.

**Conclusion** Our technique negates the errors in planning and execution. It also helps in reducing the learning curve associated with the control of osteotome making it beneficial for a novice plastic surgeon. It is particularly useful for small humps, and can be used both in open and closed rhinoplasty making it a very good alternative to conventional hump reduction techniques.

## Introduction

Dorsal hump reduction is one of the most commonly performed maneuvers in rhinoplasty. 1 Various techniques have been described for dorsal hump reduction. The most frequently used technique for small humps (< 3 mm) is filing with a diamond rasp. For larger hump reductions, a double-guarded osteotome is used to incrementally excise the bony hump in a caudocranial direction. 2 3 Lowering the nasal dorsum requires precise preoperative planning and execution of the plan. The margin for error in the management of hump is minimal, especially when the hump is subtle. Excessive excision can lead to too concave a profile. On the other hand, an inadequate excision of the hump may retain the deformity after surgery. The exact amount of lowering of nasal profile is usually subject to the surgeon's judgment rather than an objectively measured element. We present a technique of dorsal hump reduction that allows the exact amount of reduction with precision using surface measurements.

Why is there a need for the “precision technique”?

The conventional methods of planning and execution of hump reduction are prone to errors.

**Fig. 1:**
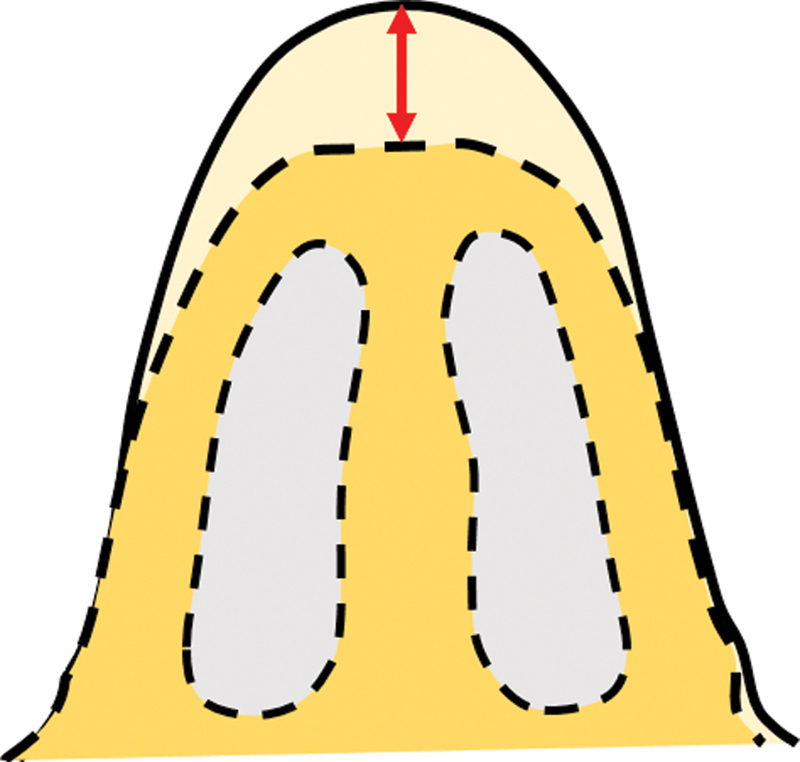
The difference between the existing contour (continuous line) and proposed contour (broken line) is shown by an arrow.

**Fig. 2:**
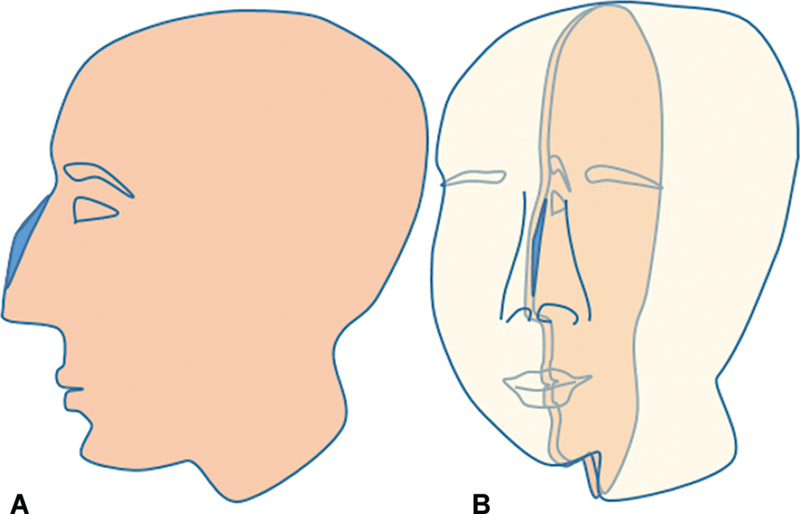
( **A** ) Planning of a hump reduction on two-dimensional photograph and ( **B** ) the three-dimensional representation of the hump.

1. *Possibility of error in planning* : The nasal projection is a matter of perception, as it cannot be measured on the body surface. The hump is a perceived amount of extra projection of the dorsum beyond the desired profile line. This is essentially a dimension/measurement along the sagittal plane and hence, lies in the core of the body and not on the surface ( Fig. 1 ).
  For any distance to be precisely measured, both the endpoints must lie on the surface. Since the posterior point of the hump dimension lies within the core, the precise amount of excess projection cannot be determined. A measurement can only be made when the three-dimensional face is flattened to two dimensions, like in a life size photograph in profile view.
However, the estimate planned on a flat photograph ( Fig. 2A ) is in fact a core dimension ( Fig. 2B ) and cannot be projected back on the nose while executing the operation. Second, getting a life size profile photo without tilt or rotation of the head and with exact magnification may be difficult. Thus, the amount of the profile line to be lowered is always a matter of judgment.
  The differential thickness of the skin–soft tissue envelope also necessitates some adjustments as the contour of the degloved dorsum is different from that of the framework draped with tissues. On a degloved dorsum, the hump appears accentuated. If the desired contour of nondegloved nose is fashioned on degloved framework, the same contour would be maintained only if the covering envelope is of uniform thickness. However, this is not the case. As the envelope is thinner in the keystone area, the contour of the profile line would lower, once the envelope drapes on the framework. Thus, reduction cannot be based on isolated appearance of bare framework, and may lead the surgeon to err on the side of excessive reduction. As the new profile is decided by the combined projection of framework and the envelope, thinner skin in this area must be taken into account and accordingly reduction of bare framework must be lesser to compensate for the lack of skin thickness. After the planned resection the profile may not look satisfactory on the degloved framework. The combined projection of framework and envelope should look satisfactory.
  An attempt to recreate the planned contour on bare dorsum is bound for failure.
  Skeletonization of the framework gives an appearance of a flattened dorsum.
This may lead the surgeon to reduce the dorsum less than planned. 4 Rather than plotting the desired contour on the bare dorsum, plotting the measured amount of reduction can minimize the errors. However, as estimated core measurements cannot be plotted, we use surface measurements to decide the amount of reduction.
2. *Possibility of error in execution* : In the conventional technique, a double-guarded wide osteotome is driven through the nasal bones in a caudal to cranial direction. This maneuver is prone to errors, as the cranial endpoint of the osteotome is difficult to judge and any error in the angulation of osteotome can alter the amount of the excised tissue.
3. *Possibility of error in estimating the variation of skin envelope* : The approach of incremental reduction by rasping; temporarily draping the envelope and judging the contour of the profile line is also prone to error. There is a difference between the contour achieved on table and the one that appears a few days postoperatively. On table the reduction may appear adequate, yet, postoperatively the dorsum projects a bit more than desired, resulting in residual hump.

To overcome the errors, we propose two innovations: first, a method to precisely plan the amount of excision based on real surface measurements rather than perceived core measurements, and second, a new technique of operative execution where the bone is cut in serial (linear) perforations (postage stamp design). A 2-mm osteotome is used in transverse direction instead of the conventional double-guarded wide osteotome used in craniocaudal direction. This technique is similar to postage stamp perforations made for percutaneous lateral osteotomy and is particularly useful in patients with a small hump, where rasping alone may prove to be inadequate.

## Analysis of the Hump and Preoperative Marking

**Fig. 3:**
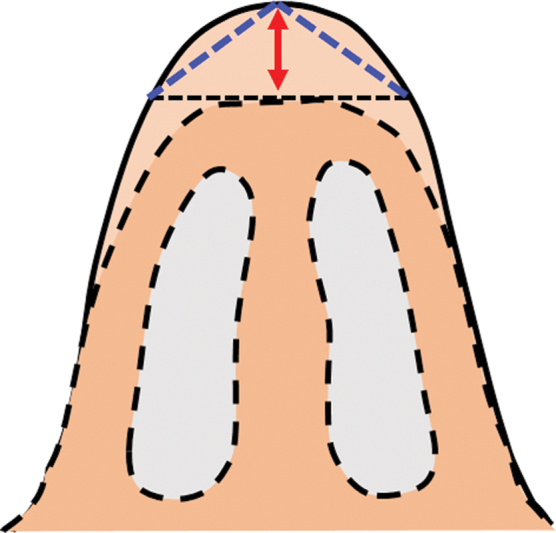
The hump can be likened to an isosceles triangle. The red arrow represents the depth (height dimension) and the blue-dotted line represents the leg dimension of the triangle.

The hump is a three-dimensional structure and can be roughly likened to an isosceles triangle on cross-section ( Fig. 3 ). The amount to be reduced (red arrow) is the difference between the actual profile (uninterrupted line) and the desired profile line (dotted line). This dimension lies along the axis of the triangle. As the axis lies in the core, it is not possible to measure this dimension. Measuring the legs of the triangle (blue lines) is simpler as these dimensions are on the surface and act as a surrogate marker. These distances are then marked on the degloved dorsum.

### Marking of the Hump Reduction

**Fig. 4:**
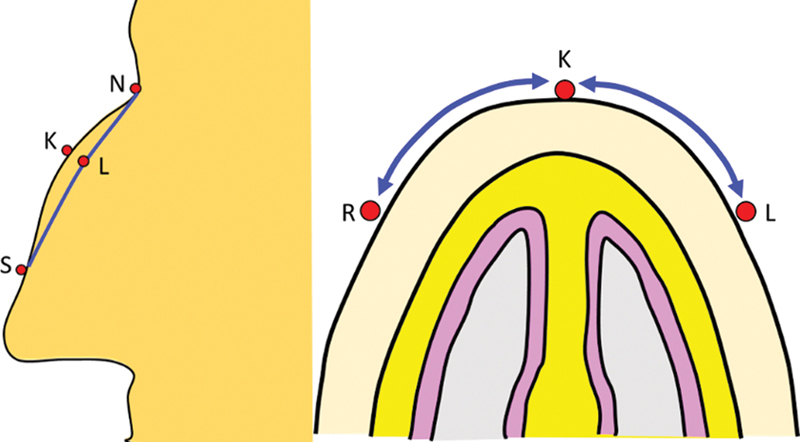
Planning of the hump reduction: The summit of the hump kyphion (K), the nasion (N), and septal angle (S) are marked. R and L represent the lateral extents of the hump.

*Preoperative* : During the consultation, the shape and extent of the hump is mapped on the patient's body, photographed, and discussed with the patient. The same marking is done again on the morning of the surgery. The patient is positioned on a chair and the nasion (N) and the anterior septal angle (S) are first marked. The existing natural midline of the nose is drawn next. This line may not be straight if there is a deformity of the dorsum. The point of maximum projection or kyphion (K) is marked on this line. The desired profile lines are then marked. Following this, the proposed points of maximum projection are marked on these lines on the lateral walls (points L and R) ( Fig. 4 ).

The points L and R are joined with the nasion (N) and the septal angle (S) creating the desired profile lines. The plotting of the desired profile line may be aided by sequentially covering the hump with a card or a flexible scale and visualizing the profile. This maneuver is performed till the profile appears desirable and the hump is masked.

The marking of the new profile line varies depending on the gender. In males, it is desirable to retain some degree of convexity of the hump. This provides a natural appearance to the nose. In contrast, a female nose should have a slightly concave appearance.

These two lines combine to form a fusiform shape that amounts to desired removal of the hump.

**Fig. 5:**
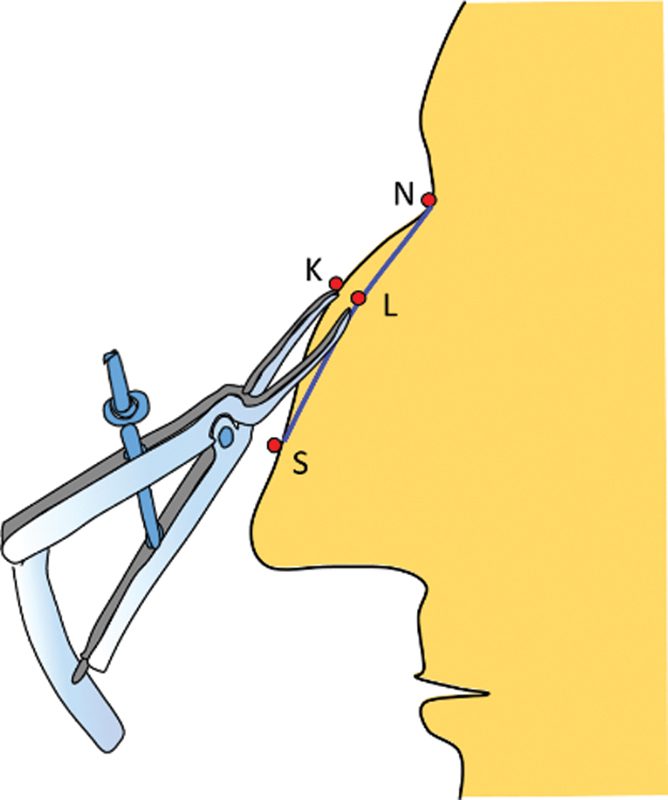
The distance KR and KL are measured with a caliper, as it gives us an aerial distance.

A caliper is then used to measure the distance K-R and K-L and dimensions are documented ( Fig. 5 ).

**Fig. 6:**
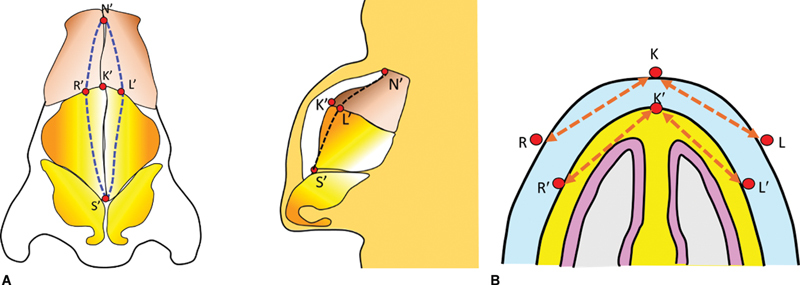
( **A** ) The five points K', N', S', R', and L' are plotted on the degloved framework. Thus, the hump is recreated. ( **B** ) The points K', N', S', R', and L' on the framework represent corresponding points K, N, S, R, and L of the envelope.

*Intraoperatively* : After raising the envelope, points K′, L′, and R′ are marked on the framework, whereby K′ is the highest point. Using the caliper, point L′ is marked on the left lateral wall whereby the distance K′-L′ is exactly the same as the distance K-L. Similarly, R′ is marked on the right side of the framework, with distance K′L′ the same as KL. Please note that the points K, L, and R lie on the surface and marked preoperatively, while points K′, L′, and R′ lie on the degloved framework and marked intraoperatively ( Fig. 6 ).

Points L′ and R′ are connected to points N′ and S′ creating framework profile lines. Hump reduction is planned along these lines. The contour of the framework profile lines is different from the surface profile lines and compensates for the differential skin thickness. As distances K-L and K-R are the same as K′-L′ and K′-R′, respectively, the amount of reduction would be precise, if done along the framework lines. Thus, this technique does not plot the contour of the profile on the framework, rather it plots the depth of excision. The crux lies in ignoring the configuration of the profile but go by the depth. The marking of the depth is along the leg surface of the isosceles triangle ( Fig. 3 ) that lies on the surface and not along the height that lies in the core.

## Materials and Methods

We conducted a prospective study in which 25 patients requiring dorsal hump reduction were included. Ten of them were males and 15 were females. All procedures contributing to this work comply with the ethical standards of the institutional guidelines on human experimentation and with the Helsinki Declaration of 1975, as revised in 2008. During the preoperative consultation, a routine nasal examination is done and standard photographs are taken. The hump is assessed and its extent is marked using the technique described above. All the operations were performed under general anesthesia. Patients were asked to fill the Rhinoplasty Outcome Evaluation (ROE) questionnaire preoperatively and 1 month postoperatively.

## Operative Technique

If the patient requires no maneuver other than hump reduction, then it can be executed by closed approach. However, if it is to be combined with other complex maneuvers, an open approach is preferred.

**Fig. 7:**
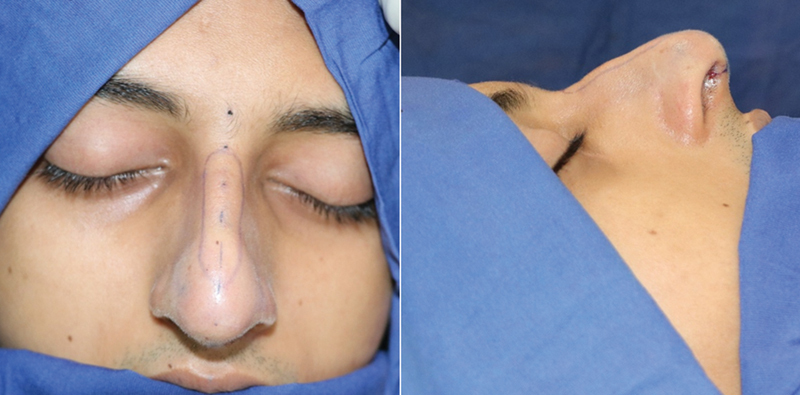
Marking of the hump on the envelope after preparation and scrubbing.

The new profile lines are marked and their distances from the natural midline are measured using a caliper preoperatively as described. The incision lines are infiltrated with lignocaine and adrenaline solution (1 in 2 lakhs) ( Fig. 7 ).

In open rhinoplasty, the columellar inverted V incision is used and the skin and soft tissue envelope is raised to expose the dorsum.

In closed rhinoplasty, an intercartilaginous incision is made and extended on to the septum.

**Fig. 8:**
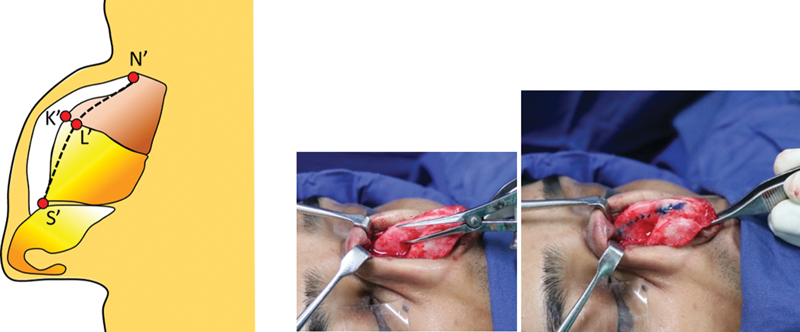
Marking of the hump on the degloved framework. The distances measured earlier on the envelope are marked on the framework.

Once the framework is exposed, we use the perichondro-periosteal flap technique 5 to elevate the flap of upper lateral cartilage perichondrium and nasal bone periosteum. This results in complete skeletonization of the dorsum. After the dorsum is exposed, the highest point on the framework is marked as K′. Using a caliper the points R′ and L′ are marked on the framework, keeping the distance K′L′ the same as KL and the distance K′R′ the same as KR. The marking of the excision lines is now replicated on the framework by joining the points R′ and L′ to N′ and S′ ( Fig. 8 ).

### Management of the Cartilaginous Hump

**Fig. 9:**
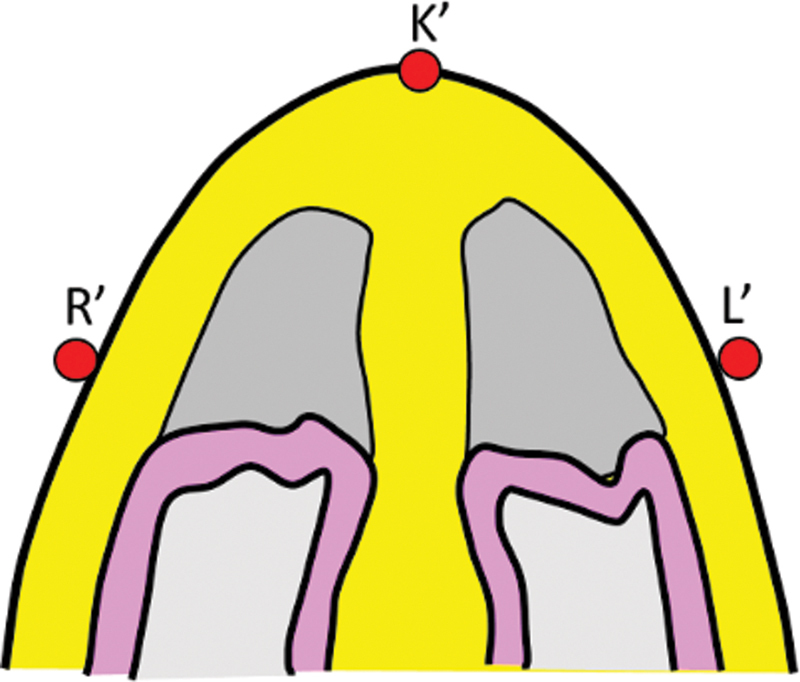
Execution of cartilaginous hump reduction. Submucoperichondrial tunnels are created as shown.

The lines on the framework give us the extent of excision only on the surface (lateral walls). We need to mark the extent of reduction in the core (septum) as well. Submucoperichondrial tunnels are created on the undersurface of the hump ( Fig. 9 ). The upper lateral cartilages are separated from the septum taking care to not damage the lining.

**Fig. 10:**
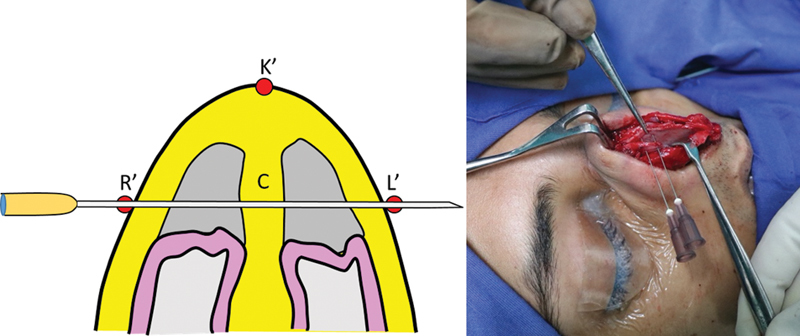
Use of methylene blue stained needles to quantify the amount of septal reduction based on points L' and R'.

To quantify the reduction of the cartilaginous septum, two straight needles stained with methylene blue are driven in a transverse direction from the right line to the corresponding point on the left line across the cartilaginous septum. This maneuver stains the septal cartilage at the desired level of excision ( Fig. 10 ).

The stained needle perforations are located and are excised using either a surgical blade or straight cartilage scissors. The upper lateral cartilages are then partially incised along the marked profile line. At a later stage the excess parts of the upper lateral cartilages are folded medially and used as spreader flaps.

### Management of the Bony Hump

**Fig. 11:**
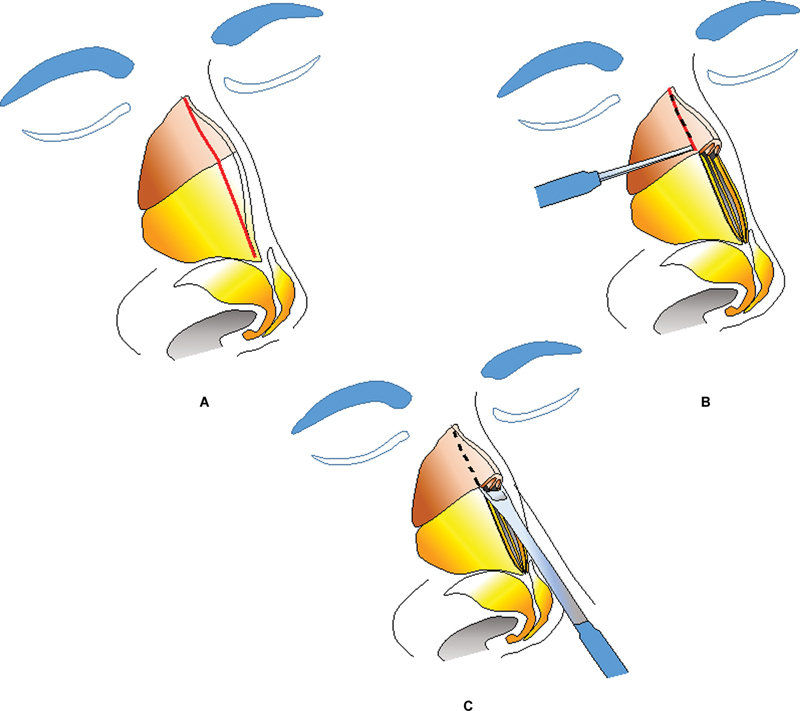
Postage stamp technique. ( **A** ) Proposed marking of the hump. ( **B** ) Excised cartilaginous hump and use of 2 mm osteotome to make perforations on the desired bony excision line. ( **C** ) Once the hump is defined bilaterally, double-guarded osteotome is used in the craniocaudal direction to excise the hump.

For the excision of the bony hump, we use a lateral approach. This is in contrast to the traditional caudocranial approach. Along the bony marking of the profile line ( Fig. 11A ), a 2-mm osteotome is used in transverse direction to make serial perforations in a postal stamp fashion ( Fig. 11B ). These perforations are similar to the linear perforations of the external lateral nasal osteotomy. As similar perforations are made on the opposite side, the guiding line for the definitive step of excising the bony hump has been defined.

A double-guarded osteotome is then driven in caudocranial direction just like the conventional technique ( Fig. 11C ). A carefully driven osteotome will travel along the path of least resistance and the postal stamp perforations guide the osteotome along the precise inclination, to convert the interrupted line to a continuous one. So any error of excessive or inadequate reduction is eliminated.

After removal of the bony hump, lateral and transverse osteotomies are done to close the open roof. The spreader flaps of the upper lateral cartilages are turned in and secured with sutures. The skin–soft tissue envelope is draped back on the dorsum. Any visible deformities are addressed. The dorsum is palpated for irregularities. Most of the dorsal irregularities are removed using a rasp. Minor irregularities are managed by the placement of perichondro-periosteal flap back to its place. It is easier to follow the marking on the lateral surface to make multiple perforations as against the caudocranial approach. The conventional caudocranial approach is a relatively blind approach, and it is likely that the plane of proposed excision may be missed resulting in underexcision or overexcision.

## Results

Twenty-five patients were operated over the past 2 years using this technique. The amount of hump reduction performed ranged from 2 to 12 mm. Four out of 25 patients required rasping to smoothen the profile. None of the patients required any additional maneuver or technique like addition of diced cartilage. All the patients were followed up at 1 month and again at 1 year. They were asked to fill the ROE questionnaire at subsequent visits. No major complications were encountered and on 1-year follow up all patients were satisfied with their results.

## Case Examples

**Fig. 12:**
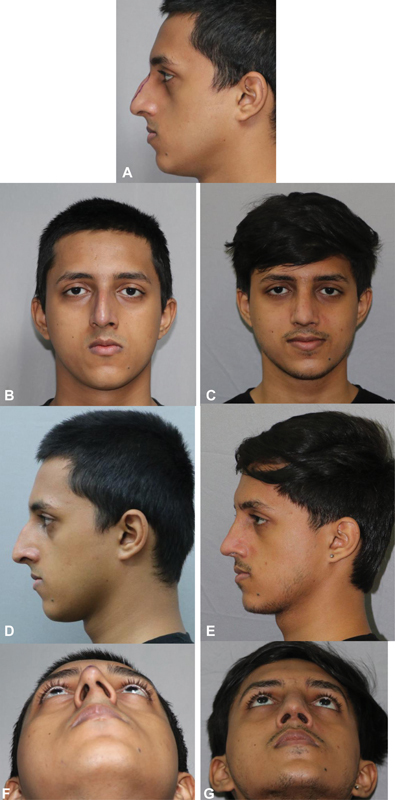
( **A** ) The marking of the hump to be excised. Patient was operated using the precision hump reduction technique and the result was aesthetically pleasing on the frontal view. rontal view, ( **B** , **C** ) profile view showing the hump,(D) which is not visualized on the postoper- ative view ( **E** ) The improvement in the worms view is also evident ( **F** , **G** ).

A 19-year-old male presented with a dorsal hump ( Fig. 12A, B ). The measured amount of reduction was 6 mm. The patient had a minimal deviation ( Fig. 12C ).

The hump was excised using the technique described. Osteotomies were also performed and the slight deviation of cartilaginous septum was corrected ( Fig. 12D ). Postoperatively, the dorsum appeared aesthetically pleasing on profile view ( Fig. 12F ). The postoperative profile view matched the preoperative proposed profile and the dorsal aesthetic lines were created on the frontal view and the tip appeared symmetrical ( Fig. 12E ). The ROE questionnaire score improved from 7/24 to 21/24.

**Fig. 13:**
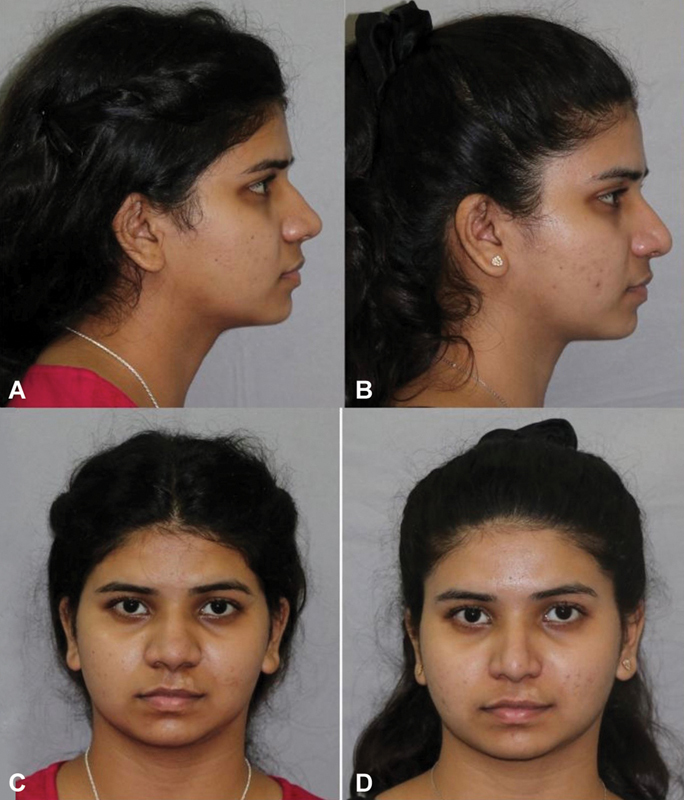
Our technique is particularly useful for patients with a small hump. ( **A** ) The hump excision was planned and executed as described above and the result shows a smooth dorsum ( **B** ). The frontal view shows improvement with defined dorsal aesthetic lines ( **C** , **D** ).

A 21-year-old female presented with external nasal deformity comprising of broad nose and tip ( Fig. 13A ) and dorsal hump ( Fig. 13B ). We performed dorsal hump reduction, alar wedge resection, tip plasty and sutures, and osteotomies. Her postoperative results were aesthetically pleasing ( Fig. 13C, D ).

**Fig. 14:**
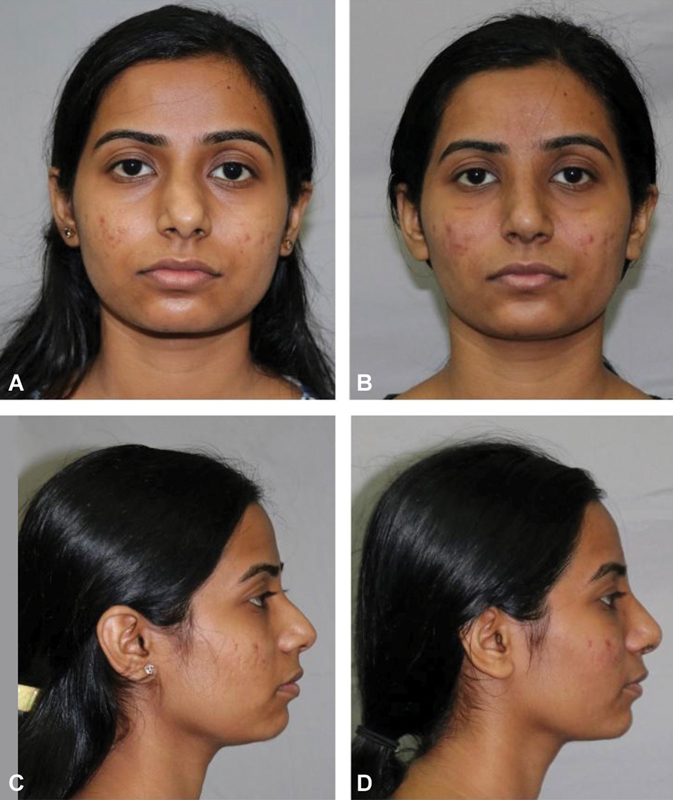
A young lady with a deviated nasal septum. ( **A** ) The operative plan included septal correction, osteotomies, and hump reduction. The postoperative frontal view shows no obvious deviation ( **B** ), and a predominately cartilaginous hump ( **C** ), and a feminine dorsum ( **D** ).

A 26-year-old lady presented with a deviated septum and a small hump ( Fig. 14A–C ). The septum was corrected and hump was reduced. The postoperative appearance had a straightened septum and the hump was corrected ( Fig. 14D–F ). The operative plan included septal correction, osteotomies, and hump reduction by precision technique.

**Fig. 15:**
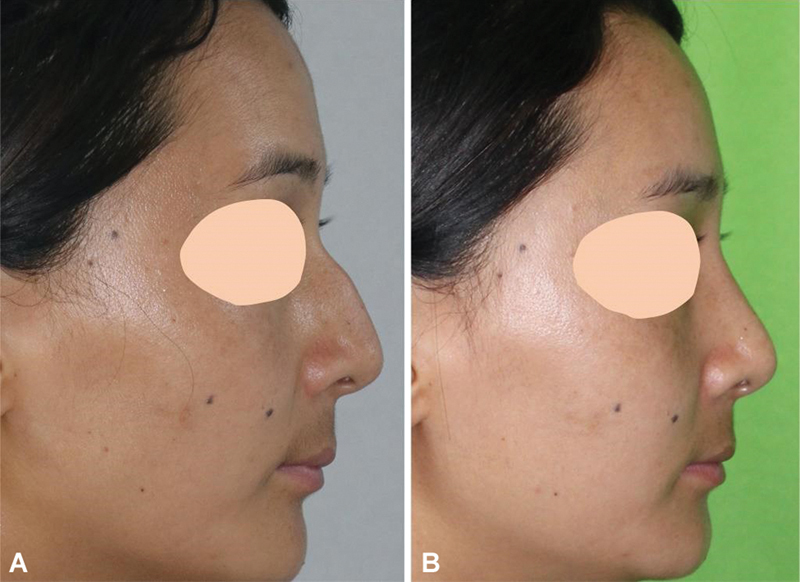
A young lady with a predominately bony hump. ( **A** ) The postoperative appearance shows an aesthetically pleasing dorsum ( **B** ).

A 24-year-old lady with a predominantly bony hump ( Fig. 15A–D ) underwent hump reduction by our technique. The profile appeared aesthetically pleasing postoperatively.

## Discussion

The need to devise the precision technique was felt considering the shortcomings of the existing techniques of dorsal hump reduction. This technique aims to negate the errors in planning and execution and also compensates for the change in contour with respect to differential skin thickness. We have used this technique with satisfactory results with the final profile matching our proposed profile line and with no major complications so far.

This technique is extremely beneficial for novice plastic surgeons as it gives control over the excision of small to large dorsal humps due to the perforations created by osteotome throughout the proposed resection. The conventional caudocranial approach is a relatively blind approach with no control and precision. The lack of judgment can result in over- or underreduction of the hump as well as deformities like open roof deformity, saddle nose, inverted V deformity, etc. 6 7 Moreover, factors like previous nasal fractures, brittle, or thinner bones influence the pathway of osteotome. 8 It is easier to follow the marking on the lateral surface to make multiple perforations as against the caudocranial approach. Thus, our technique helps in reducing the learning curve associated with the control of the hump reduction with an osteotome, hence can be performed safely and with precision by a young rhinoplasty surgeon.

The technique has proven to be of great use in cases where the hump is relatively small. Considering the precision in planning and execution, the chances of inappropriate excision are reduced.

In the modern world of rhinoplasty there are several high-precision emerging techniques like piezoelectric devices. Our technique can be highly useful in cases where there is nonavailability of such instruments. With the use of 2 mm osteotome and proper planning, precise results can be achieved.

The only disadvantage is a slightly longer time taken to make the transverse osteotomies (postage stamps). However, the optimal results outweigh this disadvantage.

Our study is subject to one limitation, which is the limited number of cases.

## Conclusion

In our dorsal hump reduction technique, we analyze the preoperative digital photographs of the patients. However, there is lesser emphasis on photograph analysis and more on marking on patient's body. We precisely calculate the amount of hump reduction using real surface measurements. Our limited experience suggests that the postage stamp precision technique can be used in both closed and open approach with equal efficacy, making it a very good alternative to conventional hump reduction techniques. This technique is particularly useful even for small humps in which the margin of error is minimal. Hence, we recommend that this technique should be routinely employed for reductions of hump.
